# A low-cost, long-term underwater camera trap network coupled with deep residual learning image analysis

**DOI:** 10.1371/journal.pone.0263377

**Published:** 2022-02-02

**Authors:** Stephanie M. Bilodeau, Austin W. H. Schwartz, Binfeng Xu, V. Paúl Pauca, Miles R. Silman

**Affiliations:** 1 Department of Biology, Wake Forest University, Winston-Salem, NC, United States of America; 2 Center for Energy, Environment, and Sustainability, Wake Forest University, Winston-Salem, NC, United States of America; 3 Department of Computer Science, Wake Forest University, Winston-Salem, NC, United States of America; Swansea University, UNITED KINGDOM

## Abstract

Understanding long-term trends in marine ecosystems requires accurate and repeatable counts of fishes and other aquatic organisms on spatial and temporal scales that are difficult or impossible to achieve with diver-based surveys. Long-term, spatially distributed cameras, like those used in terrestrial camera trapping, have not been successfully applied in marine systems due to limitations of the aquatic environment. Here, we develop methodology for a system of low-cost, long-term camera traps (**D**ispersed **E**nvironment **A**quatic **C**ameras), deployable over large spatial scales in remote marine environments. We use machine learning to classify the large volume of images collected by the cameras. We present a case study of these combined techniques’ use by addressing fish movement and feeding behavior related to halos, a well-documented benthic pattern in shallow tropical reefscapes. Cameras proved able to function continuously underwater at deployed depths (up to 7 m, with later versions deployed to 40 m) with no maintenance or monitoring for over five months and collected a total of over 100,000 images in time-lapse mode (by 15 minutes) during daylight hours. Our ResNet-50-based deep learning model achieved 92.5% overall accuracy in sorting images with and without fishes, and diver surveys revealed that the camera images accurately represented local fish communities. The cameras and machine learning classification represent the first successful method for broad-scale underwater camera trap deployment, and our case study demonstrates the cameras’ potential for addressing questions of marine animal behavior, distributions, and large-scale spatial patterns.

## Introduction

Understanding species’ habitat use and biodiversity is central to ecology and conservation of marine and terrestrial ecosystems, though these data are often some of the most difficult to gather. Terrestrial camera trapping is a growing field and a technique widely applied in global biodiversity monitoring [1]. Camera traps are remotely activated cameras that usually trigger based on the difference between background radiation and a warm-bodied animal passing through the sensor’s field. They have been used primarily to study mammals and birds, although the field is now expanding to include some ectotherms [2].

Camera traps are used to study species richness [2] and the distribution, abundance, habitat use, and behavior of wildlife around the world, with many studies surveying more than one species at a time [3]. Most camera traps are small, relatively inexpensive, and often deployed in groups or networks over a wide area for months at a time. They are typically less invasive and more reliable than comparable observation techniques [4].

Analogous techniques for monitoring underwater species face several operational challenges, chiefly the attenuation of infrared radiation in water, which renders a standard commercial camera trap unlikely to trigger underwater, except at very close range [5], and the rigors of operating in the marine environment where water intrusion and algal and faunal fouling are persistent issues, especially in nearshore systems [6]. The fact that most fishes are ectotherms further complicates use of the traditional heat-triggered infrared sensor technology used in most terrestrial camera traps. Far-red illumination invisible to most fishes provides one potential alternative to infrared [7], although far-red light still attenuates over short distances underwater. Given the ability of sound to propagate well underwater, acoustic techniques provide another possible alternative to infrared sensing [5]. Acoustic cameras [8,9] have been used in the past to image sharks and other fishes in low-light, turbid environments, replacing light-based imaging entirely [10] for species that are morphologically distinct enough to be differentiated in this manner [11]. This suggests that specific acoustic cues could also be used to trigger conventional optical cameras [12], although this would have limited applications outside of underwater camera trapping.

Due to the power requirements of active triggering (far-red light, sonar) and recording methods, most current underwater fish monitoring and measurement techniques are either limited by short battery life and operate on the scale of hours, as with baited remote underwater video (BRUV) and similar short-term recording devices [7,13–17], or require a tethered, external power source [18,19]. Because of this, the spatial extent, number of cameras, and duration of monitoring for marine systems are vastly smaller in scope than terrestrial efforts (compare the work of Williams et al. [7] and Siddiqui et al. [20] to the TEAM [21] or Snapshot Serengeti [22] datasets). Even long-term underwater monitoring with an external power supply may be limited to recording images or video during daylight hours [18] due to the difficulties of avoiding reflected particulate matter in underwater images taken at night with direct illumination. Without a side-mounted flash or otherwise indirect lighting, both white light and infrared images may be obscured by the illumination of biotic and abiotic particulates suspended in the water column, although optical backscatter filters and other multispectral image processing techniques can improve image quality in low-light and/or turbid environments [23,24]. Continuous illumination may also attract fishes and other marine organisms, depending on the color of the light [25]. Thus, long-term underwater observations are limited with regard to both power and nighttime illumination, and available solutions (e.g., batteries, specialty lighting rigs) are expensive. Both monetary costs and logistics can limit deployment of underwater cameras in remote locations without access to external power or consistent upkeep.

A related challenge faced by both terrestrial and marine camera traps is the time cost related to processing and analyzing large photosets obtained from multiple cameras over the course of months or years [22]. Applications of computer vision are growing in the field of camera trapping. They have the potential to reduce time cost [2] and have already been successfully applied to the extensive Snapshot Serengeti camera trap dataset [22] as well as images and video frames from cabled observatories or short-term marine cameras [18–20,26–28], long-term GUARD1 cameras on marine floats [29,30], and autonomous underwater vehicles [31].

Here we present a simple design for an affordable, long-running, autonomous underwater camera based on an existing commercially-available terrestrial model. We outline the deployment of our Dispersed Environment Aquatic Cameras (DEACs) across a 270 km^2^ tropical reefscape, our testing, and our subsequent analysis of the 100,000+ images obtained by implementing a deep convolutional neural network (CNN) technique for image classification. To demonstrate both the efficacy of our design and the value of long-term unmanned underwater observations to marine ecology research, we present a case study in fish feeding behavior as it relates to a well-documented benthic pattern at Lighthouse Reef Atoll, Belize of sand halos around coral heads [32,33].

Halos consist of a bare sand or lightly vegetated border surrounding a coral patch or similar underwater structure that separates the reef from surrounding dense vegetation (i.e., seagrass or algae). The heightened grazing observed inside these halos could be due to a landscape of fear [34], where herbivores are afraid to venture past a threshold distance from the reef due to predation risk [35]. However, this threshold of fish density could also be due simply to the natural dispersion of grazers and other fishes as they venture farther from the reef, which serves as a central aggregating structure for many species [36–38]. We proposed that if a strong landscape of fear is in effect, herbivorous species observed and photographed in the halo will never venture out into the surrounding seagrass, except perhaps when traveling in schools. However, a simple drop-off in fish density with distance would still result in the occasional grazing reef fish being seen by our cameras, and if reef fishes are food limited rather than predator limited, they should forage widely in the seagrass, which is a preferred food [39]. Therefore, regular detection of reef herbivores out in the seagrass over multiple months could be taken as evidence refuting the landscape of fear at Lighthouse Reef.

## Methods

### Design

#### Camera selection

Cuddeback Silver Series scouting cameras, model 1231 (Cuddeback, Green Bay, WI, USA), were chosen for their compact size, their relatively high 20 megapixel (MP) image resolution, and their time-lapse function, which allows images to be taken at pre-programmed time intervals and certain light levels without requiring the use of additional video or motion-triggered image settings. Since infrared motion triggering does not function well in the underwater environment, the presence of a time-lapse option was critical and is increasingly available in terrestrial trail cams. The cameras were programmed to take one 20 MP image every 15 minutes whenever ambient light levels were high enough to allow for color photography without flash, using the “Day” setting. Nighttime images and video and all infrared sensor-triggered images and video were disabled to conserve both power and memory space because initial field tests showed that few or no additional usable images were captured using the infrared, low-light “Night” setting.

All cameras deployed throughout the study period were synchronized to record photos on the hour and every 15 minutes following to ensure that images from different cameras and sites were captured at the same time of day and under the same local conditions. The 15-minute interval was chosen to balance the need for regular observations of reef and seagrass communities that may include transient fish species and the constraints of storing and processing thousands of high-resolution images collected by multiple cameras over a months-long deployment. The ability of this interval to adequately capture the community composition and species present at a given reef was validated using in-person diver surveys (described below).

Memory cards were 32 GB SanDisk (Western Digital Corporation, Milpitas, CA, USA) or Kingston (Kingston Technology Corporation, Fountain Valley, CA, USA) microSD cards with adapters, capable of holding over 25,000 20 MP color images each. Each camera required eight Energizer Ultimate Lithium AA batteries (Energizer Holdings, Inc., St. Louis, MO, USA), which provided enough power for over five months of continuous function under the settings described here.

The total cost of each camera, including the batteries and SD card, came to just $125 per unit with the housing (discussed below). The use of pre-built commercial trail cameras, which are designed for energy efficiency over long deployments, significantly reduced both material and energy costs, relative to constructing a similar camera from scratch using components like a Raspberry Pi (Raspberry Pi Foundation, Cambridgeshire, UK) and GoPro (GoPro, Inc., San Mateo, CA, USA), Canon (Canon, Inc., Ota City, Tokyo, Japan), or Sony (Sony Corporation, Minato City, Tokyo, Japan) cameras, as used in previous underwater camera applications [7,15,16,20,26].

#### Housing construction

Two housings of different materials were tested in the field, both based on commercially available junction box enclosures. Housing 1, based on item DS-AT-1217-1 available from “Saipwell” (Saip Electric Group Co., Ltd, Wenzhou, China), has thinner walls (2.4–3.9 mm) made of an unspecified plastic. The top secures with specially-shaped plastic screws and it contains an O-ring-like insert made of foam. Housing 2, based on model ML-47F*1508 from Polycase, Inc. (Avon, OH, USA), is a thick-walled (3.5–4.0 mm) design made of polycarbonate resin secured with stainless steel screws and a silicon rubber gasket. Both housings had a 2-inch diameter hole drilled in the faceplate and a 3-inch disk of 1/8 inch thick acrylic epoxied to the opening with MarineWeld (J-B Weld Company, Atlanta, GA, USA) to act as a window. Since both housings used identical acrylic windows and contained the same cameras, images collected using each design were indistinguishable; the chief difference between the housings was their pressure tolerance and leakage at depth. Each housing cost approximately $30 for all components (included in the total price given above). Ablative antifouling boat paint was applied to the housing exterior, excluding the back and acrylic window, of a subset of cameras with Housing 2 to reduce biofouling.

Cameras were programmed, armed, and packed inside their housings with cardboard spacers. Housings were sealed with Star brite marine silicone sealant (Star brite, Fort Lauderdale, FL, USA) around the seam of the enclosure lid. Silicone sealant was also used to reinforce the edges of the epoxy seal around the lens window, both inside and outside.

#### Field installation

**Fig 1:**
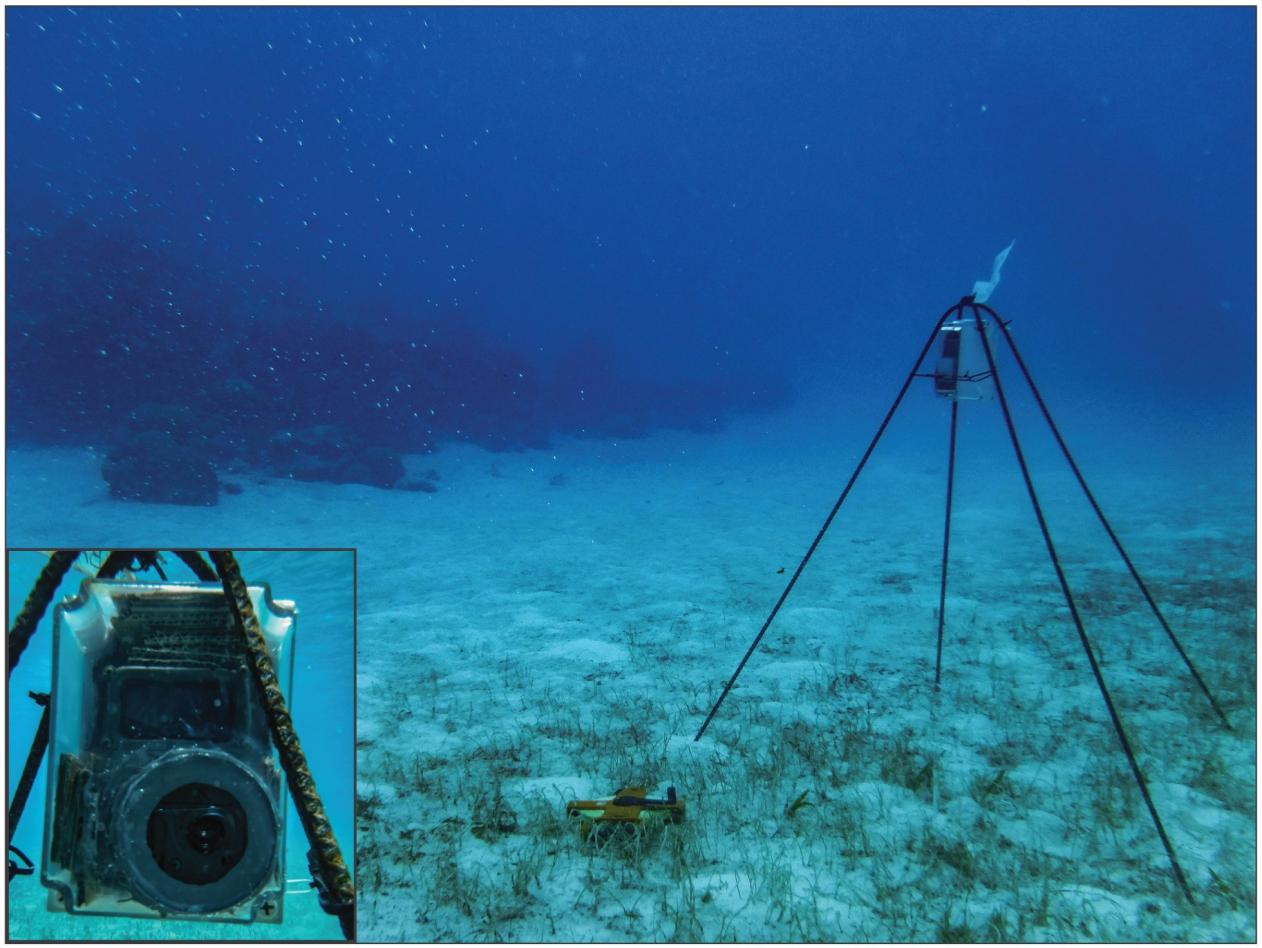
DEACs deployed on four-legged rebar stands in the field. Plastic cable ties attached to the camera housing and to the legs were used to secure buoyant cameras in housings below the crossed rebar at the top of each stand. Bottom left inset: A detailed front view of a camera in Housing 1 underwater. Reprinted from [39] under a CC BY license with permission.

Cameras were secured to four-legged bent rebar stands with plastic cable ties threaded through mounting holes pre-built into the housings (Fig 1). Due to their buoyancy, camera housings were placed underneath the crossed rebar forming the top of each stand and secured laterally to the four legs, which were sunk into the sediment to keep the stands upright. Lightweight plastic or polystyrene buoys were tethered to a small subset of cameras and stands located in particularly shallow water (2 m or less) to prevent collision by boats.

### Deployment

#### Study system

Lighthouse Reef Atoll off the coast of Belize is primarily a shallow (1–8 m depth) lagoon environment, dominated by scattered patch reefs interspersed with a mosaic of seagrass, macroalgae, and sand [40]. Although relatively isolated from the mainland, the atoll is subject to heavy local fishing pressure for conch, lobster, and certain fish species. A small no-take marine protected area (MPA) surrounds the island of Half Moon Caye in the southeastern corner of the atoll. Because of its shallow benthos, wide variety of benthic cover types, and spatial variation in protected status, Lighthouse Reef provides an ideal location to test our DEACs under a variety of conditions. The shallow marine grazing system of Lighthouse Reef and similar Caribbean locations is in many ways analogous to terrestrial grazing systems like the African savanna [41], where camera trap networks have been effectively deployed for years [22].

To structure our testing and demonstrate the utility of the DEACs for addressing ecological questions at large spatial scales, we organized our deployments around detection of herbivorous fishes grazing in coral reef halos at Lighthouse Reef. Grazers at Lighthouse Reef are mostly large parrotfishes (Scaridae) and surgeonfishes (Acanthuridae). These diurnal herbivores readily consume seagrass when it is provided to them within the halo surrounding a reef [39]. We conducted nighttime observations and grazing assays (see [39]) to ascertain that nocturnal grazers like urchins were not particularly active at our sites. We deployed our DEACs in a paired design that allowed us to monitor the reef and halo as well as the surrounding seagrass or macroalgae at multiple sites to determine whether these herbivorous reef fishes regularly venture into the surrounding seagrass to feed and are simply more abundant in the halo due to its proximity to the reef or if they are constrained to the reef and adjacent halo by predation (a landscape of fear).

#### Spatial arrangement

Cameras were deployed in pairs at 21 patch reef sites within Lighthouse Reef Atoll. Deployments occurred asynchronously between March 2018 and March 2019 (S1 Fig), and a total of 28 individual cameras were used across all deployments, with some cameras deployed multiple times. The majority of sites were occupied by cameras between June and August 2018, with the longest deployments lasting over 5 months (August 7, 2018 to January 12, 2019). DEAC sites were distributed evenly inside and outside of the Half Moon Caye Natural Monument MPA in the southeastern corner of the atoll. Seven sites (3 inside the MPA, 4 outside) featured predominantly algal bottom cover; the rest were in areas surrounded by seagrass, primarily *Thalassia testudinum*. Patch reef sites for camera deployment were chosen via random point placement using satellite imagery and a depth map of the atoll (courtesy of the Carnegie Airborne Observatory), which allowed sites to be evenly stratified across depths from 2–7 m.

**Fig 2:**
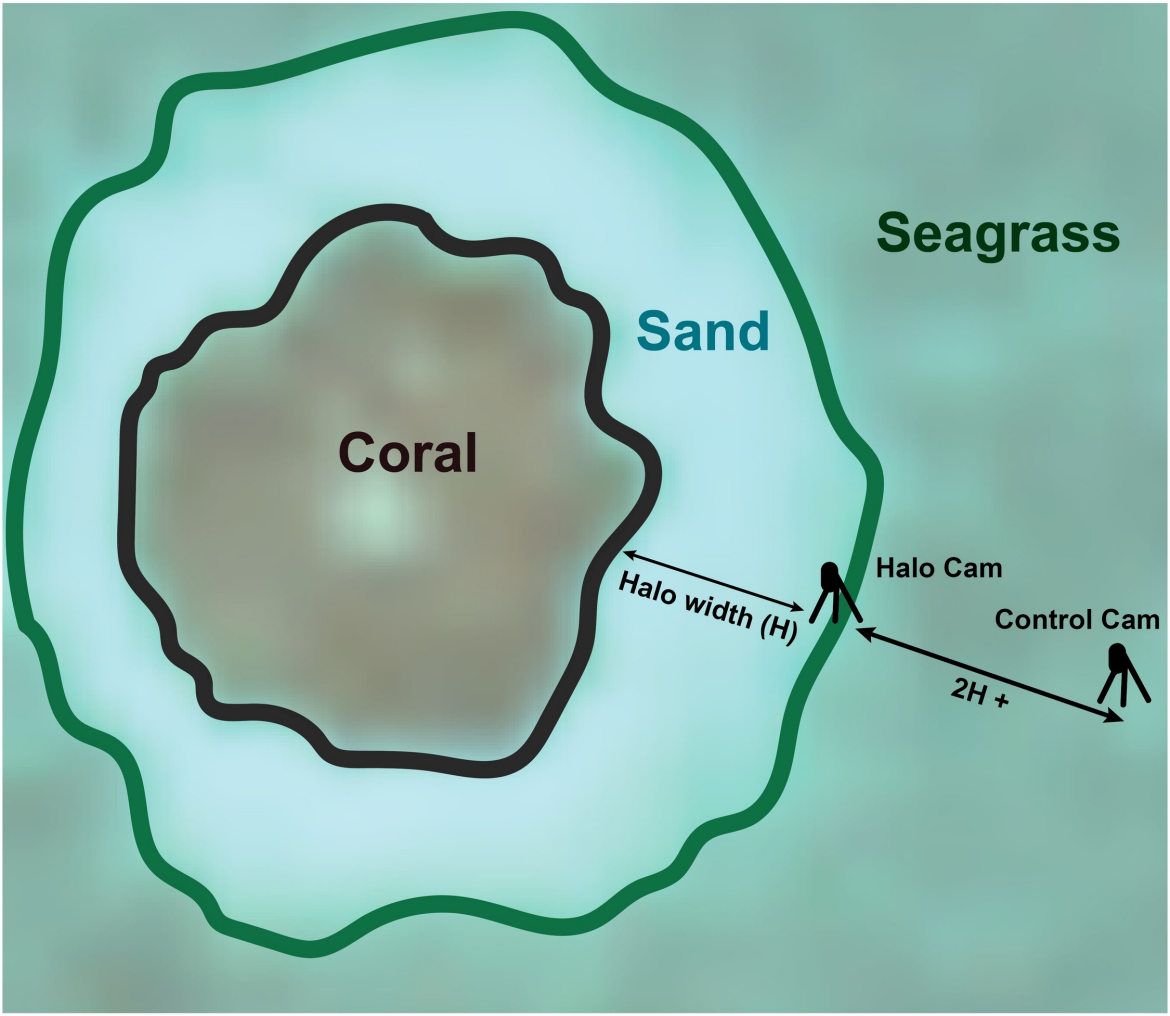
Camera arrangement at a patch reef site. One camera was placed on the edge of the halo facing in toward the reef (“Halo Cam”), while the second (“Control Cam”) was placed twice the halo’s width away in the surrounding seagrass or algal cover, in line with the first camera. Modified from [39] with permission.

Sites deeper than 4 m were initially avoided due to leakage of Housing 1 past this depth, although depths of up to 7 m (maximum depth required in the study) were successfully achieved with Housing 2, which was used for initial deployments in March 2018 and all deployments from August 2018 onward. Cameras were deployed in sets of two, one camera located at the edge of the sandy halo surrounding a patch reef and the other located at least twice the halo’s width away from the edge in the surrounding seagrass or algal benthic cover (Fig 2). In the case of particularly narrow halos, “control” cameras were placed a minimum of 15 m from the halo edge. This allowed control cameras a view of the same bottom cover (seagrass or macroalgae) as that adjacent to the halo but placed them well beyond the fish density thresholds observed by Layman et al. [38], while also accounting for the possibility that larger halos could represent reefs with larger or farther-ranging fish populations. “Halo” cameras had a relatively wide field of view, as is typical of terrestrial trail cameras, which included both the halo in front of them and the patch reef beyond. Both cameras were pointed toward the reef, although the edge of the halo was beyond the range of view for the grass (“control”) camera at most sites. Camera orientation with regard to compass direction was varied between sites during deployment, although both cameras at any given location were oriented in the same direction.

#### Monitoring and secondary deployments

Camera deployments occurred in stages, with the first cameras deployed in March 2018 and the last cameras collected in March 2019 (S1 Fig). The longest-running DEACs remained continuously active underwater for five months (see above) and were still operating at retrieval. Initial deployment of cameras in March 2018 included one camera at the edge of a halo and a second camera pointed at the first in order to assess whether the presence of the camera and stand had any effect on the presence or behavior of fishes. No obvious confounding effects of camera presence were noted in this initial test (neither herbivores nor large predators aggregated around the camera structure, the same species were photographed near the camera and in other parts of the halo, etc.), so doubling of cameras in this manner was discontinued for future deployments. For all subsequent deployments (March 2018 onward), the two cameras at each site were positioned to monitor different benthic environments and unable to see each other. Diver observations while cleaning and monitoring cameras in the field over the duration of this study supported our initial conclusion that reef fishes largely ignore cameras, except to occasionally eat algae off the structures, as they do with any other hard substrate in the halo. During the summer of 2018 (June to August), cameras were consistently checked every 1–2 weeks for leakage, algal overgrowth, or stand displacement. Cameras deployed at longer intervals from March to June 2018, August 2018 to January 2019, and January to March 2019 were unmonitored during these periods in order to test long-term underwater function and determine the effects of biofouling on housings and image quality in the absence of cleaning or regular adjustments.

#### Diver observations

In-water observations were conducted by a team of 2–3 divers at each camera site and at several additional locations in order to validate the cameras’ ability to detect fishes and other animals. Observations consisted of all divers sitting directly behind each camera for 15 minutes and recording the presence and abundance of all fishes and other animals observed on the reef, in the halo, or in the grass to the genus or species level, when possible. Divers faced forward toward the reef and recorded all fishes observed within the halo or on the reef itself, as this was the primary field of view of the camera. In the seagrass or macroalgae, divers again faced toward the reef and recorded only fishes that swam in front of them and the camera. Divers remained stationary for the entire observation period at each camera. Fish species and counts were determined by a consensus of all the divers present at each observation. Fish counts were recorded by divers as 1 individual, 2–3 fish, or 5 or more (coded as 1, 3, and 5), which could under-represent species for which there were many individuals at a given site. In practice, the majority of fishes observed by divers were solo or in small groups.

### Data analysis

#### Community composition

A non-metric multidimensional scaling (NMDS) analysis was run on species communities inside and outside of the halo using data from diver observations at 20 camera sites and image analyses from 18 of these cameras. For each camera, 3 images taken before, during, and after diver observation (15-minute intervals) were analyzed, for a total of 54 images. Only images from immediately before, after, and during diver observations were used for comparison in order to control for natural variation in fish communities over time, since the main goal of the comparison was to assess the ability of the cameras to capture known community composition at a given location and time. All fishes in this subset of images were identified to the genus or species level by the same divers who conducted the in-water observations. Species recorded in images from all three time points (before, during, and after divers) were pooled for each site to determine presence or absence. The NMDS used a Sørensen distance matrix calculated through the binary version of the “bray” method in vegan’s vegdist function [42] and was based on species presence or absence at each site, not abundance, to reduce the chances of “double counting” the same individual fish in successive images and to limit effects of the binned counts recorded by the divers. Fish counts across different habitat types and methods (divers and cameras) were also compared with a two-way PERMANOVA, using the adonis function in vegan [42]. To determine whether divers had any effect on fish presence, counts from images captured before, during, and after diver observations were compared using a nonparametric Friedman rank sum test. All analyses were conducted using R statistical software version 4.0.3 [43] with packages qdap [44], reshape [45], rstatix [46], and vegan [42].

#### Image sorting

Images from the larger dataset encompassing all cameras were initially named and sorted by location, time, and date using the camtrapR [47] package in R [43]. A subset of over 13,000 images were sorted by trained undergraduate student volunteers into categories containing at least one visible fish (“Fish”) and without any visible fishes (“NoFish”). To ensure consistency, all volunteers were initially trained on the same subset of ~300 images drawn from multiple different cameras and their accuracy assessed by the research team before they were assigned a larger subset of images to sort individually. Due to the nature of timed rather than motion or heat triggered photos, many images did not contain fishes.

#### Model choice

**Fig 3:**
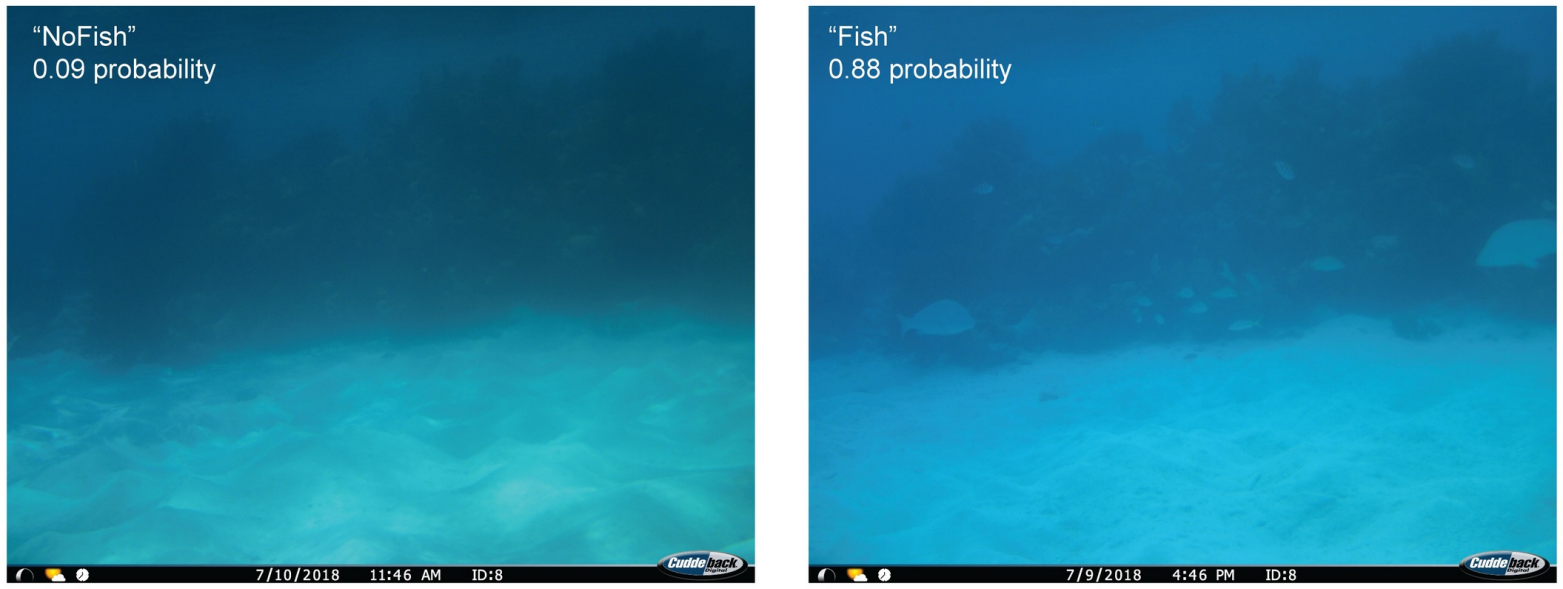
Empty (left) and fish-filled (right) images captured at the same camera site within 24 hours. The empty or “NoFish” photo was assigned only a 0.09 probability of containing a fish by our ResNet-50 model, whereas the model predicted a 0.88 probability of the second photo containing at least one fish, hence its “Fish” designation. Probabilities like these were used to sort images into “Fish” and “NoFish” categories to streamline further analysis. Reprinted from [39] under a CC BY license with permission.

In order to streamline future analyses of fish photos and reduce the need to manually classify over 80,000 images, we built and trained a CNN based on ImageNet pretrained ResNet-50 [48], a deep residual network widely used in image classification. Deep CNNs (i.e., those with many layers) are important tools for visual recognition tasks but may be hard to train because of vanishing gradients and degradation. The residual network (“ResNet”) approach solves this by explicitly allowing stacked layers to fit a residual mapping instead of directly fitting a desired underlying mapping, which enables neural network architectures to go deeper [48]. In our application, ResNet allowed us to learn Fish/NoFish representations from a noisy and relatively small dataset. We chose ResNet as our base structure because we wanted conservative results in classifying fish pictures. ResNet is powerful at preventing overfitting, making it less likely to omit pictures containing fishes. The reproducible code was implemented in Pytorch [48–50] to identify images with animals (Fig 3). It took roughly twelve hours to train the ResNet model for 50 epochs on a custom-built desktop PC with a single Nvidia RTX 2080 GPU, Intel i9-9900k CPU, and 32 GB of RAM.

#### Training and validation

Our model was trained on a sample of 10,727 images sorted by our team of trained volunteers and then run on 75,470 additional images to sort them into “Fish” and “NoFish” categories, based on the model’s calculated probability of each image containing at least one fish. To augment the training set, we preprocessed the fish images with spatial transformations, cropping and lightening variation, and used focal loss [51] as an objective function to address the problem of imbalanced labels. The model’s accuracy was validated by comparing the categories produced by sorting above and below 0.5 probability with the actual “Fish” and “NoFish” designations assigned to 2,702 images hand-sorted by trained volunteers.

#### Image analysis

Images with a “Fish” probability of 0.5 or above were used to determine the relative presence of fishes at camera sites inside and outside of the Half Moon Caye MPA, in different habitat types (reef/halo vs. seagrass/algae), and at different times of day. Detection is an imperfect proxy for actual fish presence [52], so these comparisons could represent differences in occupancy, changes in behavior, or other unknown factors. This analysis of fish detections was conducted based on the relative proportions of the total images classified that did or did not contain fishes inside and outside of the MPA and at patch reef and seagrass/algae locations, compared using the prop.test function in R [43]. The null hypothesis was that both groups in each test should have equal proportions of images containing fishes. Only predictions from images taken within the first month of deployment were used in these analyses to account for the varying degradation in image quality (and resulting increased potential for misclassification) due to biofouling at different sites. Trends in fish detection probability across different times and locations were visualized using ggplot2 [53].

## Results

### Camera performance in the field

#### Cameras and housings

Housing 1 proved vulnerable to flooding at depths exceeding 4 m and prone to leaking even at shallower depths. This appears to be due primarily to the thin nature of Housing 1’s walls and lid, which deformed substantially under pressure, breaking the epoxy seal with the lens and allowing water to enter. Housing 2 proved far more robust to pressure and had minimal leakage even at a maximum deployed depth of 7 m over multiple months. Further testing with this housing will be necessary to determine its maximum functional depth, but preliminary tests with a revised housing design have reached depths of 40 m.

The cameras themselves proved resilient to flooding and were typically still armed and taking photos when extracted from partially-flooded housings. However, many cameras recovered from flooded housings were unable to be redeployed due to lasting damage to their internal systems. Batteries were sufficient to power the cameras past the five-month extraction date of our longest deployment, with all non-flooded cameras still displaying the starting battery status of “OK” (not “LOW” or “DEAD”). Maximum water temperature measured at any of our deployment sites, which could affect battery life, was 30° C (86° F). Cuddeback advertises that their cameras can operate up to 12 months continuously with efficient battery usage. Memory cards were more than sufficient to store images on the schedule that they were collected during this period (approximately 8.5 GB of images over 5 months), suggesting that image frequency could be doubled or tripled in future deployments, assuming that processing of these additional images is sufficiently streamlined with the use of computer vision or similar techniques.

#### Biofouling

**Fig 4:**
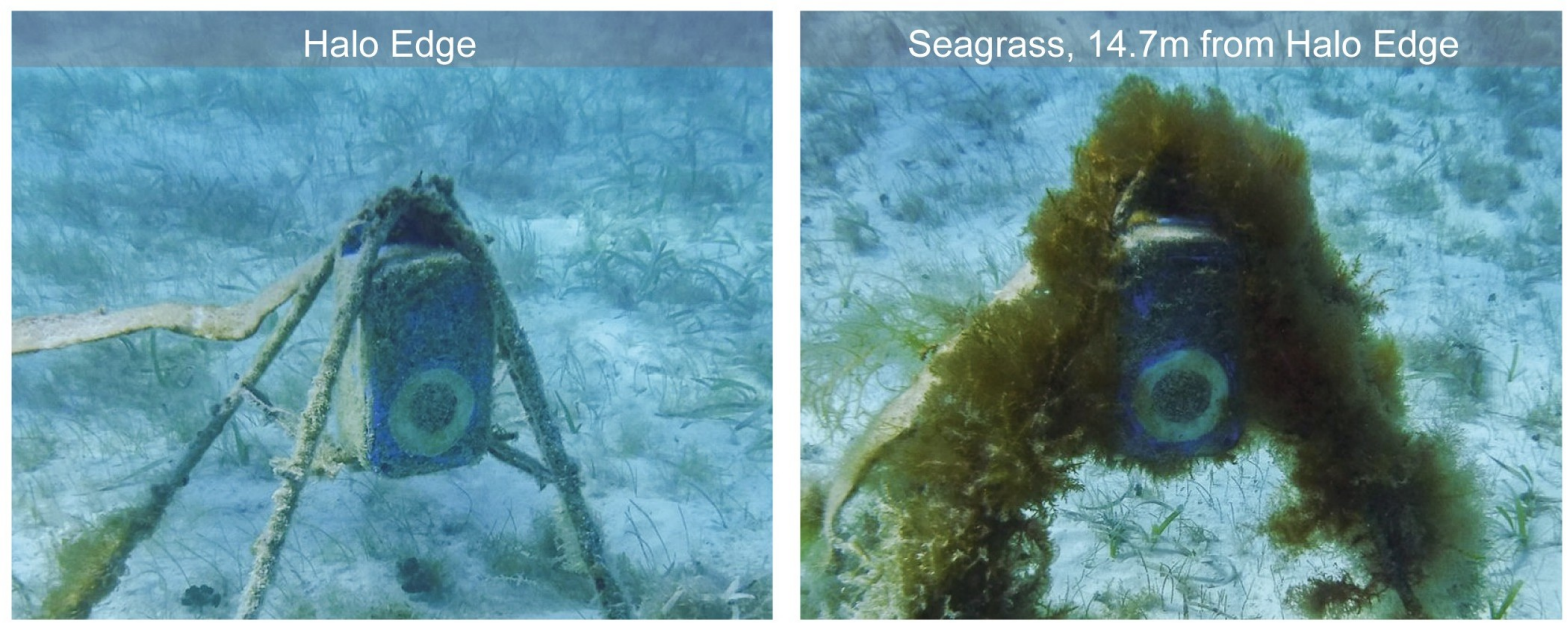
Comparison of biofouling and algal overgrowth after a month based on location. Structures were located at the edge of the halo, adjacent to seagrass (left), and in the middle of the surrounding seagrass (right). Similar results seen across all camera sites suggest that reef-based herbivores help to control algal growth both within and at the edges of the halo but do not graze heavily, if at all, on algae and related marine organisms in the surrounding algal or seagrass beds. Reprinted from [39] under a CC BY license with permission.

The most significant impediment to long-term camera function at our sites was biofouling, the growth of marine organisms over the camera housing and stands. Image quality declined rapidly due to biofouling, making fish identification impractical in as little as one month depending on site conditions (S2 Fig), with a median value of two months endurance, although imagery from some cameras remained usable up to four or five months after deployment. This biofouling decreased both fish detections in images and the ability of our model to accurately classify images taken by biofouled cameras (S3 and S4 Figs). Algal grazing by fishes was an important factor reducing fouling on cameras and stands placed near patch reefs, while cameras placed in seagrass or algae beds away from patch reefs were overgrown with algae over the same deployment period that halo cameras remained relatively unobstructed (Fig 4). The addition of antifouling boat paint to the camera housings before the August 2018 deployment appeared to be only a minor deterrent to organisms growing on the housing in general and did not prevent the camera lens window from being almost completely obscured 2–3 months after deployment. In addition to biofouling, camera function was also limited by reduced visibility at certain sites, which varied based on local turbidity and light availability, and by depth.

### Image classification and accuracy

Of the 130,621 images collected, 88,899 were deemed usable (68%) based on a visual examination, despite some level of biofouling in many of these. Images were included in the model if the researchers could still visually distinguish the shapes of fishes, the reef, and other underwater features despite the biofouling. The ability to identify individual species was impaired in the most heavily fouled images we included, but this was not a primary goal of our particular model. Since the determination regarding image quality was made by humans, it is possible that the threshold at which we considered an image unusable was not the optimal cut-off point for the processes used by the model to evaluate image content. Our ResNet-50 model had an overall accuracy of 92.5% when classifying these images into “Fish” or “NoFish” categories, with higher accuracy in identifying empty or “NoFish” photos, likely due to the increased number of these in the training dataset (Table 1). The training and validation sets of manually classified images together made up approximately 15% of the total usable images obtained from the cameras.

**Table 1:** Accuracy of model predictions.

True Image Designation	Correct Predictions	Incorrect Predictions	Percent Accuracy (%)
*Fish*	444	165	72.9
*NoFish*	2057	36	98.3
**All images**	**2501**	**201**	**92.5**

Accuracy is determined by comparing the computer’s prediction for each image with the actual label of “Fish” (image contains at least one fish or similar animal) or “NoFish” (image is empty) assigned to the photo by a team of trained volunteers. The model was trained on 10,727 images classified by volunteers and then validated with an additional 2,702 human-sorted images, the results of which are shown in this table. Prediction accuracy is higher for “NoFish” (empty) images, likely due to the higher proportion of this type of image in both the training and validation datasets.

### Community composition

#### Comparison with diver observations

**Fig 5:**
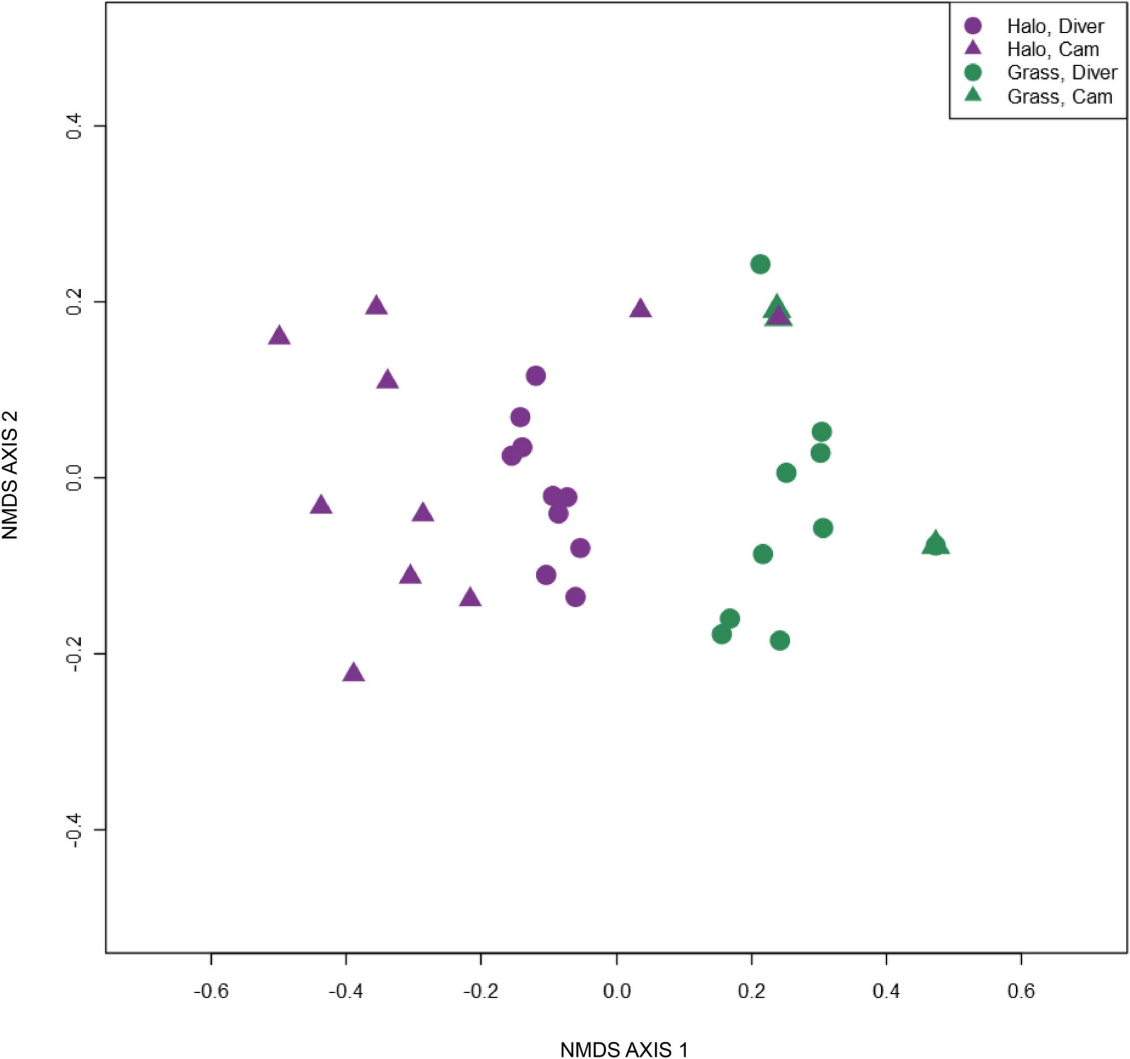
Non-metric multidimensional scaling (NMDS) based on presence/absence data. Points represent 20 individual site observations by divers or 3 pooled images from 18 given sites and are colored purple for halo/reef cameras and dark green for seagrass or algae cameras. ANOSIM of this NMDS output found a significant difference between halo/reef (purple) and seagrass/algae (green) communities (R = 0.68, p<0.001). Dots represent diver observations, while triangles represent camera images. Both diver and camera observations reflect the same clear difference between fish communities on the reef and those in the seagrass. See S5 Fig for a stressplot of this NMDS.

Large compositional differences were identified between the halo/reef and seagrass/algae fish communities, which were reflected in both the diver species observations and those from camera images (NMDS; Fig 5), although each 45-minute set of three images consistently contained fewer fishes than were observed by divers during a 15-minute period at the same site. An ANOSIM conducted on the NMDS output showed a significant difference between the halo/reef and seagrass/algae communities (R = 0.68, p<0.001), which was reflected in both the camera and diver species observations. The PERMANOVA revealed significant differences between diver and camera methods (F = 3.92, df = 1, p = 0.002) and between habitat types (F = 7.15, df = 1, p<0.001), as well as an interaction between the two (F = 2.31, df = 1, p = 0.028). Overall, there was no significant effect of diver presence (before, during, or after) on fish counts from the cameras (Friedman rank sum test p = 0.15, Kendall’s W = 0.09).

#### Fish distributions

**Fig 6:**
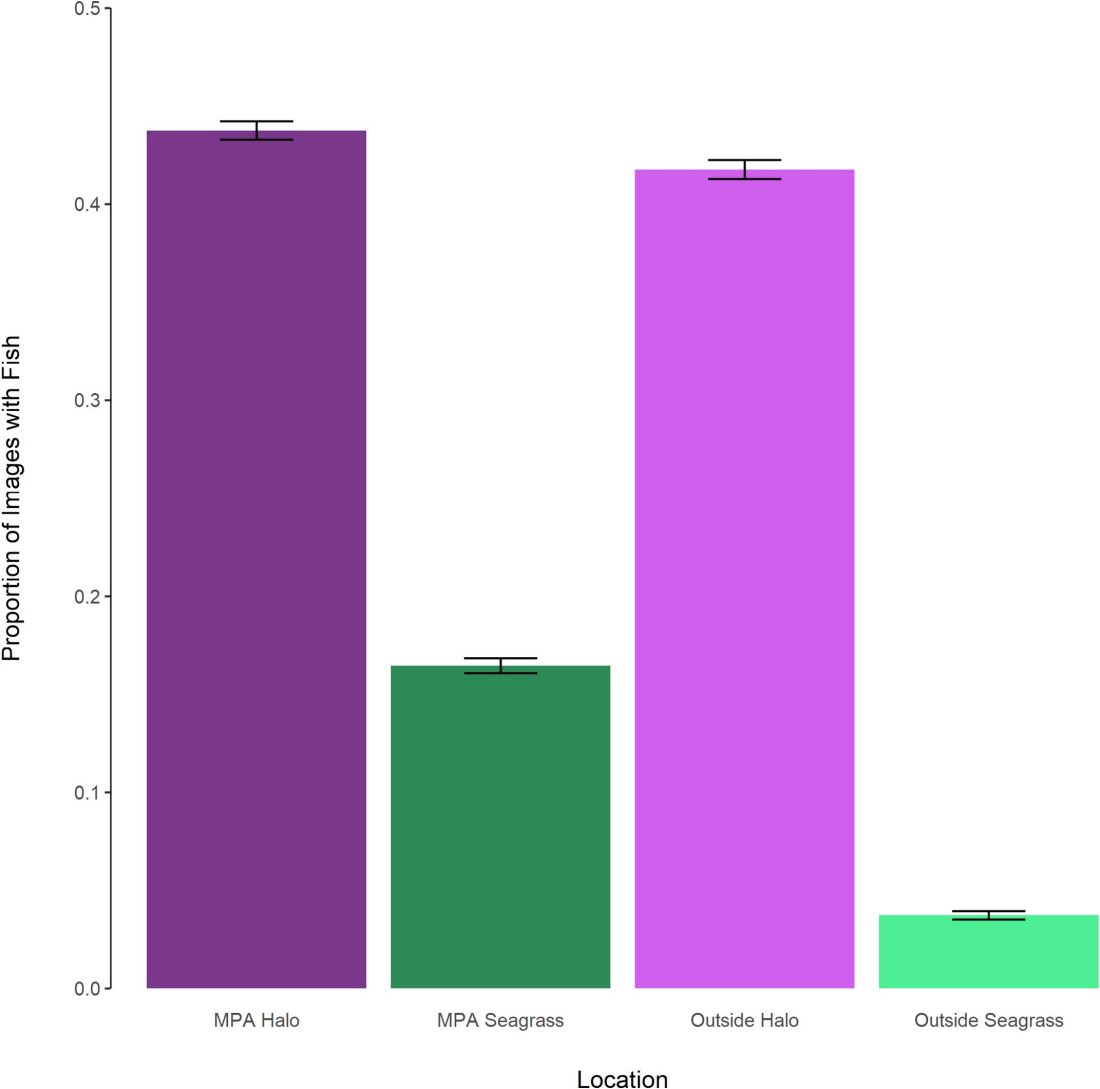
Proportion of images containing fishes at halo and grass/algae sites inside and outside of the Half Moon Caye MPA. Halo sites are represented in shades of purple, seagrass/algae sites in shades of green. Each bar is a proportion based on the n for that treatment. Error bars represent ± 1 standard deviation from the binomial distribution, scaled by the respective n for each treatment.

**Fig 7:**
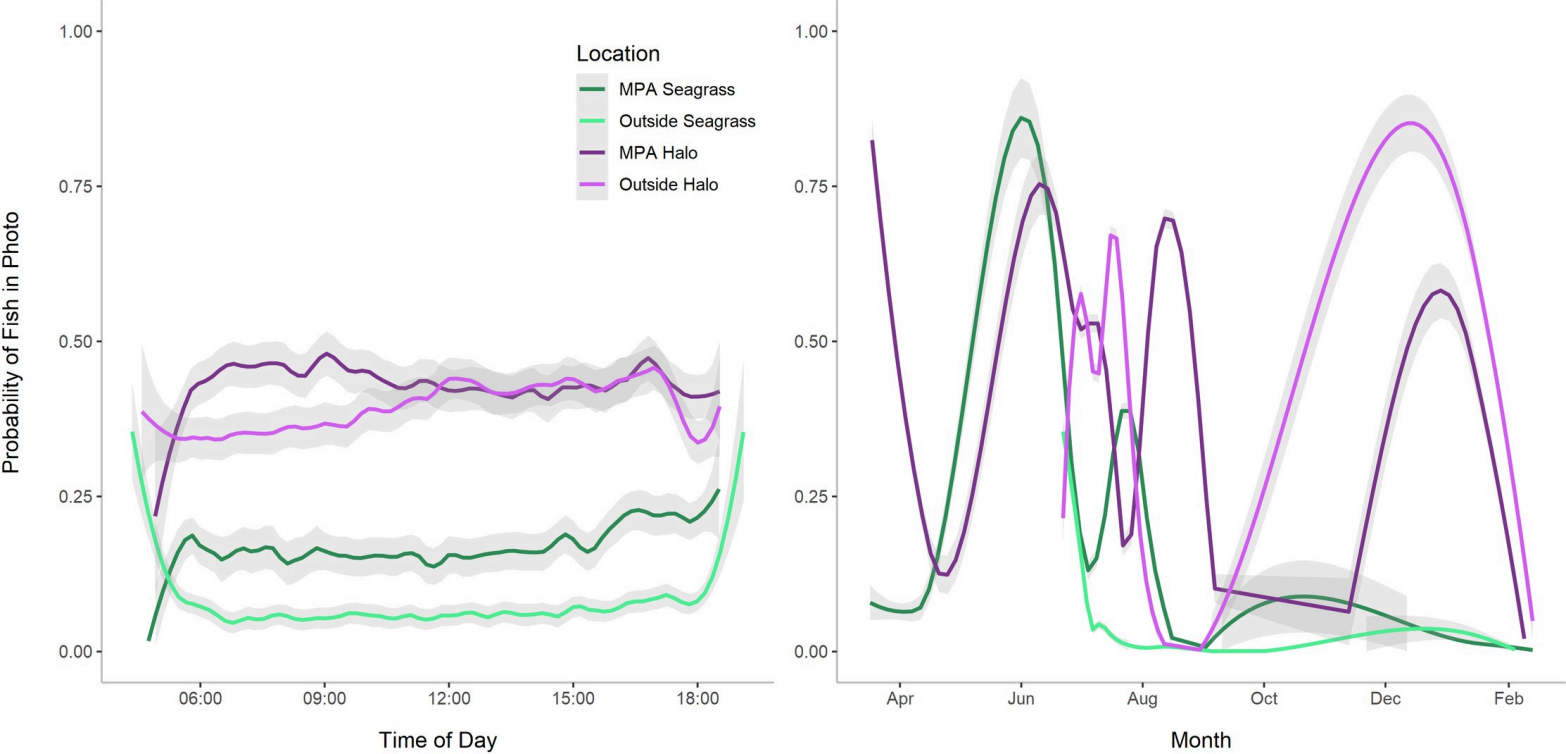
Time series showing smoothed data from 40 different cameras deployed asynchronously between March 2018 and March 2019. (A) Fish probabilities for individual images in halo/reef and seagrass/algae habitats by time of day. Probabilities ranged from 0 to 1 for this dataset, but smoothing visually compresses the highest and lowest probability values on the graph. (B) Fish probabilities for images by date, showing variation over the course of one year at Lighthouse Reef Atoll. Trend lines for all groups were generated using the geom_smooth function with method “loess” in ggplot2 [53]. Light gray shading represents 95% confidence intervals.

The fish detection counts based on camera data revealed that the proportion of images with fishes was 32% higher in halos compared to seagrass or algae camera locations (χ^2^ = 4892.2, df = 1, p<0.001). Fish counts were 38% higher in algae environments than in seagrass (χ^2^ = 5298.3, df = 1, p<0.001), which could represent the actual presence of animals in these habitats or might reflect differences in the ability of humans and human-trained computer vision to distinguish small fishes in the denser seagrass cover. Protected status also had a smaller but significant effect, with a 6% increase in the proportion of photos with fishes within the MPA, compared to outside the MPA (χ^2^ = 174.09, df = 1, p<0.001). Analysis of camera locations relative to both benthic cover (i.e., reef/halo or algae/seagrass) and protection status (inside or outside MPA) revealed that fish detection is always higher in halos than in seagrass or algae communities at Lighthouse Reef, and the number of fishes in each community type is relatively higher inside the MPA (Figs 6 and 7). Fishes were consistently more abundant around reefs and halos than in surrounding vegetation. However, there may also be a seasonal effect, with fewer fishes detected in seagrass environments during the fall and winter (October through February), while fish detections in halos increased during this time (Fig 7).

A preliminary analysis of diver observations from 20 camera locations as well as an additional 60 images from those sites did not contain a single instance of an herbivorous reef fish (surgeonfish and all adult parrotfish except for *Sparisoma radians*) outside of a halo. Predatory fishes, including barracuda (*Sphyraena barracuda*) and multiple species of jacks (*Caranx* spp.), were seen in both the reef/halo and seagrass/algae environments at different sites. Snappers (*Ocyurus chrysurus*, *Lutjanus* spp.) were seen primarily on the reef.

## Discussion

### Camera performance

#### Deployment duration and function

Underwater camera traps proved to be energy-efficient, durable, and capable of producing large volumes of quality images representative of the fish communities at their locations. The DEAC camera trap design is a reliable, cost-effective, and easy-to-implement solution allowing the expansion of terrestrial camera trapping techniques to shallow marine environments. The set-up can easily be used to vastly expand the capability of BRUVs and associated techniques [13–15] and can also provide marine observations over periods of months. In addition to being long-term and more scalable, un-baited camera traps such as these may be more accurate than BRUVs in their estimation of population and community composition [54] and allow the use of random encounter models for occupancy that are widely used with terrestrial camera traps [52].

Camera placement and orientation is an important consideration when deploying DEACs in the field, and the ideal placement may vary with geographic location, season, target time of day, water quality, and shading from local structures like patch reefs or docks. The cameras have no observable effect on the behavior of marine animals and may therefore be used to document species that typically evade divers. Even cameras that are “cleaned” by herbivorous fishes in the halo do not appear to attract any more attention than natural patches of algae or coral rubble. They are also capable of remaining underwater at depth for long periods and recording high volumes of photo or video observations—our longest running cameras were deployed for 5 months, captured over 7,500 images each, and still had battery life to extend to a year. The exceptional duration and the ability to capture vast numbers of images, and to automatically recognize photos with fishes (see below) gives the capability to not only provide a continuous record of rare species behaviors or visits by transient species that divers would miss or only observe by chance, but also, for the first time, to record changes in behavior, relative abundance, and migrations across seasons and lunar phases. DEACs are flexible in their ability to record both photo and video at a variety of time intervals suitable for addressing different ecological and behavioral questions.

#### Comparison to existing methods

DEACs offer significant advantages over existing underwater camera options for remote, shallow marine environments, as well as surveys by divers. While the longer time interval chosen for our extended duration field tests did not capture as many individual fishes in each image as divers recorded during the same 15-minute period corresponding to a single photo, analysis of synchronous diver and camera observations at different sites demonstrated the ability of both methods to capture differences in community composition between distinct benthic environments (Fig 5). These compositional differences also support the placement of the halo and control cameras at each site, suggesting that the cameras were separated enough to capture these different adjacent community types while remaining close enough to control for local site conditions. The only group of animals observed by divers that were not well captured by DEAC images were schools of roving juvenile fishes and small parrotfishes that camouflage well within seagrass and algal environments and are best detected through movement, which may have contributed to the interaction between habitat type and observation method detected by the PERMANOVA. The inability of the camera images and corresponding CNN to identify these fishes may be due to a combination of more limited image quality in these turbid environments and the inability of human sorters (on whose data the CNN was trained) to distinguish these small fishes later in a static image. Since these species are neither threatening predators nor significant grazers on seagrass or foliose macroalgae [55,56], their detection or inclusion in fish community composition was relatively unimportant for addressing the question used here to assess DEAC utility. However, for applications where detection of these species or similarly small or cryptic organisms is important, use of short video clips in place of still images (an option readily enabled with the DEAC design) could increase the ability of both humans and machines to detect these animals through motion. Because of their long deployment time, the DEACs captured differences inside and outside of the MPA that were not revealed by the diver observations, as well as changes based on time of day and season (Fig 7), which are hard to quantify with a limited number of diver surveys.

The current duration of camera operation is over an order of magnitude longer than the operational duration achieved by similar non-tethered underwater cameras [7,20], even with biofouling seriously impairing image quality after 1–2 months (S2 Fig). To the best of our knowledge, these are the first such underwater camera traps to be affordable (<$200 per unit), power-efficient (therefore deployable over the span of months) and also self-contained, without the challenges imposed by a surface-tethered external power source, which typically precludes deployment in remote regions and at multiple sites over large areas.

The most closely related marine camera application to the DEACs is GUARD1 [29,57,58], an autonomous camera trap also operating on a time-lapse and for similarly long durations (48,298 images over 5.5 months [58]), which uses on-board image recognition and classification to detect gelatinous zooplankton in open waters. While GUARD1 is also a “low-cost” solution, the price of just the commercial camera used in the GUARD1 system is more than the entire DEAC set-up, including housing, memory, and batteries. This makes the DEAC design more suitable for deploying networks of many individual cameras over a large area and for study designs involving pairs or groups of cameras in close proximity. DEACs are inexpensive and easy to assemble, allowing many of them to be quickly deployed to address a variety of ecological questions, while GUARD1 units are individually more costly, complicated to build, and currently engineered to address a very specific monitoring challenge. DEACs and GUARD1 are designed for different marine environments and applications, with GUARD1 optimized to detect specific zooplankton [29] and potentially also fishes [30] in open water, while DEACs are suitable for detecting fishes and other marine animals, changes in habitat or other ecosystem features, and general monitoring in shallow, nearshore benthic environments, extending the kinds of landscape-level data taken with terrestrial camera traps into the marine realm.

#### Future improvements

The greatest immediate limitation to the DEAC design is the inability to prevent or seriously reduce biofouling without periodic manual cleaning of the cameras, although the magnitude of this challenge may be location-specific. Biofouling is a common problem with unsupervised underwater monitoring equipment [59], with multiple solutions proposed to control it [6], including local chlorination [59,60], copper sheeting and mesh, UV radiation [61], and wipers [62]. Ongoing tests of camera and housing design are incorporating these methods to reduce biofouling by marine organisms and extend the functional life of the lens window to better reflect the power and storage capabilities of the camera. Preliminary tests of a similar camera design in Hawai’i suggest that the extreme biofouling observed at Lighthouse Reef is not representative of all such reef environments and may be related to especially high productivity and suspended organic matter in the water column at this location. Simple monthly cleaning of the lens with a saltwater aquarist’s sponge is another remedy if revisitation is possible.

While both the batteries and memory cards we employed were sufficient for the deployment duration and image capture frequency tested, the inability to utilize memory cards with greater than 32 GB of storage in Cuddeback cameras is problematic with a more frequent time lapse interval or in the case of short videos collected in place of single images. Therefore, optimizing and expanding both power and data storage capacity of these cameras via commercial means (cameras with larger SD storage capability) or noncommercial modifications is another potentially valuable direction for future research. For example, SD Ultra Capacity (SDUC) storage media currently affords 2 TB of storage, vastly expanding the capability of underwater camera traps for either smaller time intervals between images or multi-year deployments. Indeed, large storage capacity may obviate the need for camera triggering, making time-lapse or video with on-board object recognition (as used in GUARD1 [29]) a superior alternative, giving the ability to capture even rare or transient species while effectively using the millions of potential images generated. Design improvements currently in progress focus on reducing biofouling, extending the depth range of the camera housings to over 50 m, and implementing an external flash or other nighttime illumination.

### Image analysis

The success of our initial image sorting using the CNN ResNet-50 illustrates the power of machine learning and computer vision techniques to drastically reduce time and cost when dealing with large image sets in marine environments. Our ability to use this trained model to pre-sort images with high accuracy before attempting further analysis via manual or autonomous machine-learning based methods also reduces the cost of the most disadvantageous aspect of our timed image capture method: the number of frames with no objects of interest to the current study. Now that datasets obtained from this and similar shallow marine environments can be easily sorted to exclude non-target images, future underwater camera trap projects using a similar time-lapse method will be able to quickly remove the majority of empty frames, while retaining the ability to measure frequency of detection events and variations in fish presence by time of day, season, or other environmental variables by comparison of occupied and empty images. Expansion of the deep neural network model to focus on identification of individual species, functional groups, and/or the sizes of different individuals, as has been done in similar image analyses [16,18,20,22], will further streamline the analysis of this and related large image datasets.

Our community composition analysis also demonstrates that the 15-minute photo interval of our cameras was sufficient to capture the same community differences between benthic environments that were observed by divers in the water. This suggests that while a single image does not capture every fish active in the area during the 15-minute period it represents, the collection of images from any given site are representative of the community at that location and are likely to accurately reflect changes in species behavior, abundance, or diversity at the site over a range of time scales (e.g., daily vs. seasonal changes), perhaps more accurately than a limited series of diver observations would. Camera networks are an emerging method for monitoring fish activity and other behaviors on diel and seasonal timescales [63,64] and may reveal important information about temporal resource partitioning, predator avoidance, and other interspecific dynamics in the community [65]. Further study of camera captures vs. diver observations regarding species known to be wary of divers, either those using traditional open-circuit SCUBA or closed-circuit rebreathers [66], is an important line of future investigation to understand true reef fish occupancy and abundance.

Our analysis of the number of images with fishes collected at different groups of cameras revealed a difference undetected by the fish counts from our diver observations, showing that fishes were detected by cameras more frequently (and are therefore likely to have higher occupancy) inside of the MPA. The inability of traditional diver surveys alone to detect this difference at all reinforces the value of spatially-distributed, long-term datasets like the images collected from our cameras. The observed differences in fish detection between halo and grass/algae control sites also reinforce diver observations of both fewer fishes and a different fish community in the algae or seagrass beds away from the reef and support the results of the community composition analysis (Fig 5).

### Case study and future applications

Our observations of fish species presence across different environments are consistent with the landscape of fear explanation for heightened herbivory inside coral reef halos. We obtained a large volume of usable images from sites with a variety of depths and benthic cover types, subjected to different fishing pressures inside and outside of a local MPA, and monitored over the course of different seasons. This allows us to reasonably conclude that our observations are likely representative of fish presence and behavior at patch reef sites within Lighthouse Reef Atoll as a whole. The complete lack of grazing reef fishes observed outside of the halo region by cameras located in surrounding seagrass within 30 m or less of the halo supports the idea that a predator modification of prey fish behavior imposes real constraints on fish movement and that heightened grazing pressure adjacent to reef structures is not simply the result of fishes randomly dispersing with distance from the reef. If the latter were true, the pattern would be a simple exponential decrease in fishes as distance from the patch reef increased, which would likely be reflected in a lower (but nonzero) number of grazing fishes detected at seagrass and algae camera locations, compared to those at the halo edge.

An alternative hypothesis is that the lack of grazing fishes appearing in images taken outside of the halo is due to unequal detection of fishes between the two environments [67], possibly the result of faster or more furtive movements outside the relative safety of the halo. However, this explanation is refuted by two pieces of evidence: First, diver observations also showed no herbivorous reef fishes at grass or algal camera sites; second, other fish species that were observed by divers to move quickly through the seagrass or algal environment (e.g., bar jacks, *Caranx ruber*) appear in both halo and control camera images, indicating that cameras placed in the grass are very capable of photographing fishes moving through that environment. It is therefore more likely that the complete lack of detection of reef-based grazers outside of the halo, even though their food is in much higher supply there, is due to their total or near-total absence from this environment because of the combined lack of shelter and exposure to predators [35].

The most common large predators seen by both divers and cameras were barracuda (*S*. *barracuda*). This is based only on the 54 images used for the NMDS and anecdotal observations made in the course of sorting and processing additional images, since our CNN did not include detection of individual fishes to the species level. One of us (MR Silman) observed a barracuda preying on a large adult parrotfish, which smaller predatory fishes are unlikely to target. This demonstrates that barracuda present a real threat to even the largest herbivorous fishes in this system. Barracuda were observed in both reef and seagrass/algae environments, but the reef provides more structure (and likely protection [68,69]) to potential prey than the open seagrass beds. This could lead foraging fishes to limit their movement to within a certain radius of the reef, which is the most commonly-cited explanation for reef halos and is consistent with our observations.

This study demonstrates the value of long-term, spatially-distributed underwater camera trap observations for addressing a subtle difference in fish communities and benthic pattern generation. DEACs can be easily deployed alone or in large spatial arrays for short or extended time periods, and they are capable of recording periodic still images for long-term studies or short videos for detailed behavioral observations. Most importantly, these cameras are highly energy-efficient and require little-to-no maintenance while deployed, making them ideal for remote locations or extended observations that surface-anchored systems or commercial underwater cameras with limited battery life are not suitable for. Multiple networks of camera traps like TEAM [21] and Snapshot Serengheti [22] have been successfully deployed over large regions in terrestrial environments, and DEACs offer the option to now expand such long-term, spatially-extensive monitoring efforts to the marine realm. The use of a CNN makes processing the volume of images collected over such a long-term study a practical option, and continuing advancements in machine learning and computer vision are likely to enable further processing of similar large visual datasets in the future.

## Supporting information

S1 Fig Deployment periods for individual cameras, typically deployed in pairs at a given site. Treatment designations represent if the camera was deployed in the Half Moon Caye marine protected area (MPA) or outside (OUT), in algae (a) or seagrass (g), the site number or other site designation within that treatment, and camera location at the halo edge (H) or farther away (C),. The final number present for only certain treatments indicates which of multiple deployments at the same location this period refers to.
(JPEG)

S2 Fig Decline in fish detections due to biofouling over time for the 13 longest deployments. Deployment time in days is shown on the x-axis, while the probability of a given image containing at least one fish is shown on the y-axis, as calculated by the ResNet-50 model. Fish detection probabilities decrease over the first month, at which point the pattern becomes less clear, likely the result of inaccurate image classifications at certain sites in response to biofouling in the images. The red line represents the smoothed results of all sites combined, using the “loess” function in R package ggplot2 (Wickham 2009).
(PNG)

S3 Fig Fitted lines for fish detection probabilities over long-term reef-adjacent halo deployments. Lines were fitted with the “loess” local polynomial regression fitting function with span = 1 from R package ggplot2 (Wickham 2009). The dark line represents the smoothed results of all sites shown on this graph. Not only do fish detections decline consistently at most sites due to biofouling of the camera lens over the first month, model accuracy is clearly impaired past this point at certain sites, leading to a false increase in fish detection probability for some cameras (e.g., note the erroneously high probabilities around 80–100 days for MPATr12H, shown in blue).
(JPEG)

S4 Fig Fitted lines for fish detection probabilities over long-term seagrass or algae meadow deployments. Lines were fitted with the “loess” local polynomial regression fitting function with span = 1 from R package ggplot2 (Wickham 2009). The dark line represents the smoothed results of all sites shown on this graph. Note that fish detection probabilities are lower at grass sites, relative to reef/halo sites, and the y-axis is scaled appropriately. After the first month, fish detection probabilities erroneously rise at a couple sites, likely reflecting reduced accuracy in the ResNet-50 model’s image classifications as a result of biofouling.
(JPEG)

S5 Fig Stressplot for the NMDS shown in Fig 5 of the main text. Created using the stressplot() function in R package vegan (Oksanen et al,. 2018) [42].
(PNG)
