# Supplementary figures and images for: A low-cost, long-term underwater camera trap network coupled with deep residual learning image analysis
Source: PLoS One. 2022 Feb 2;17(2):e0263377. doi: 10.1371/journal.pone.0263377 (PMC8809566; doi:10.1371/journal.pone.0263377)

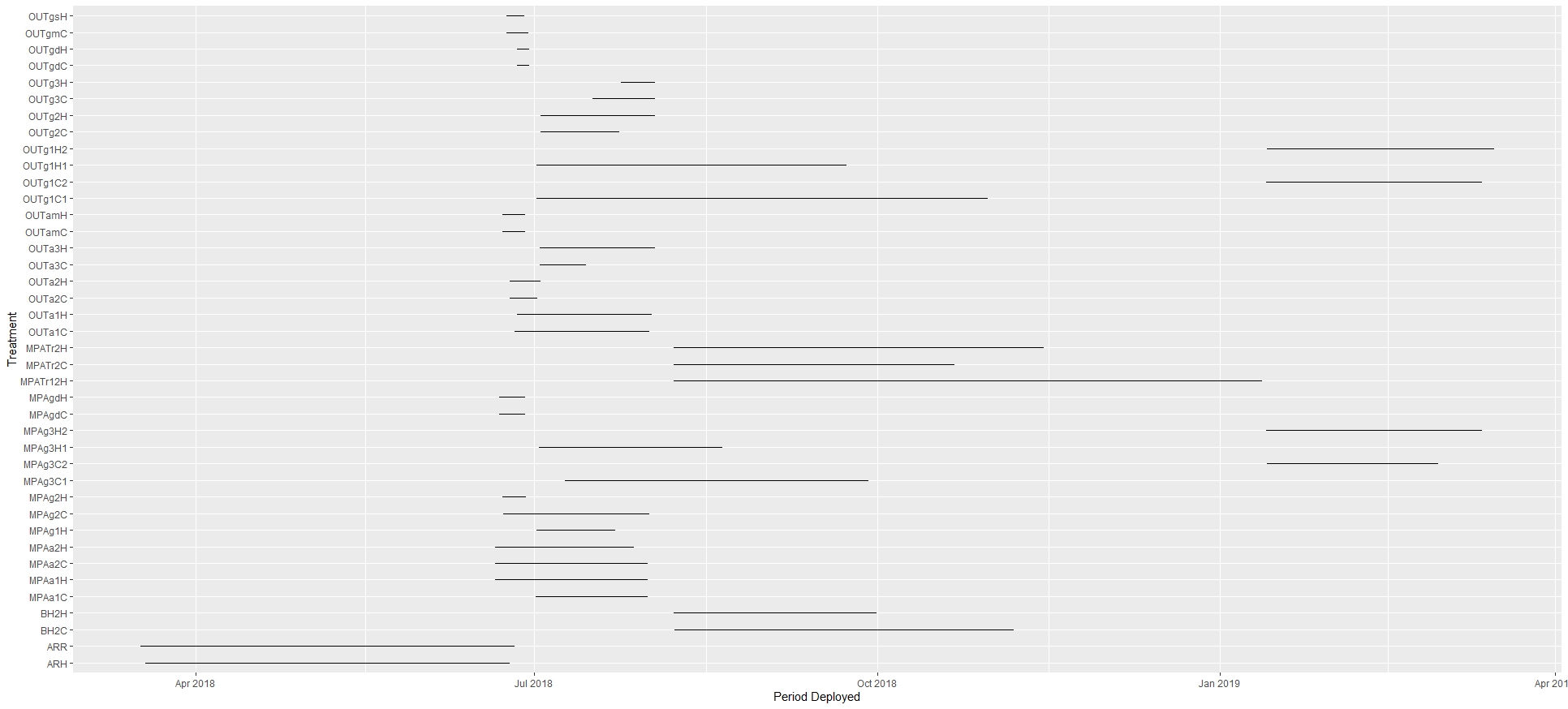

Supplement: S1 Fig — Treatment designations represent if the camera was deployed in the Half Moon Caye marine protected area (MPA) or outside (OUT), in algae (a) or seagrass (g), the site number or other site designation within that treatment, and camera location at the halo edge (H) or farther away (C),. The final number present for only certain treatments indicates which of multiple deployments at the same location this period refers to. (JPEG) [file pone.0263377.s001.jpeg]

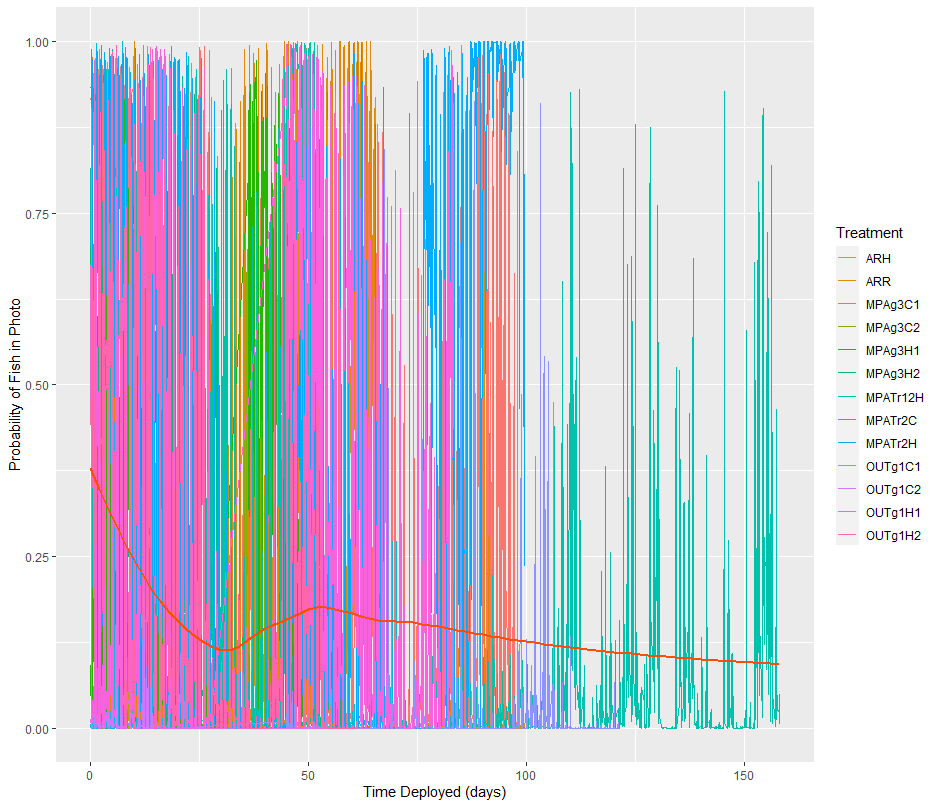

Supplement: S2 Fig — Deployment time in days is shown on the x-axis, while the probability of a given image containing at least one fish is shown on the y-axis, as calculated by the ResNet-50 model. Fish detection probabilities decrease over the first month, at which point the pattern becomes less clear, likely the result of inaccurate image classifications at certain sites in response to biofouling in the images. The red line represents the smoothed results of all sites combined, using the “loess” function in R package ggplot2 (Wickham 2009). (PNG) [file pone.0263377.s002.png]

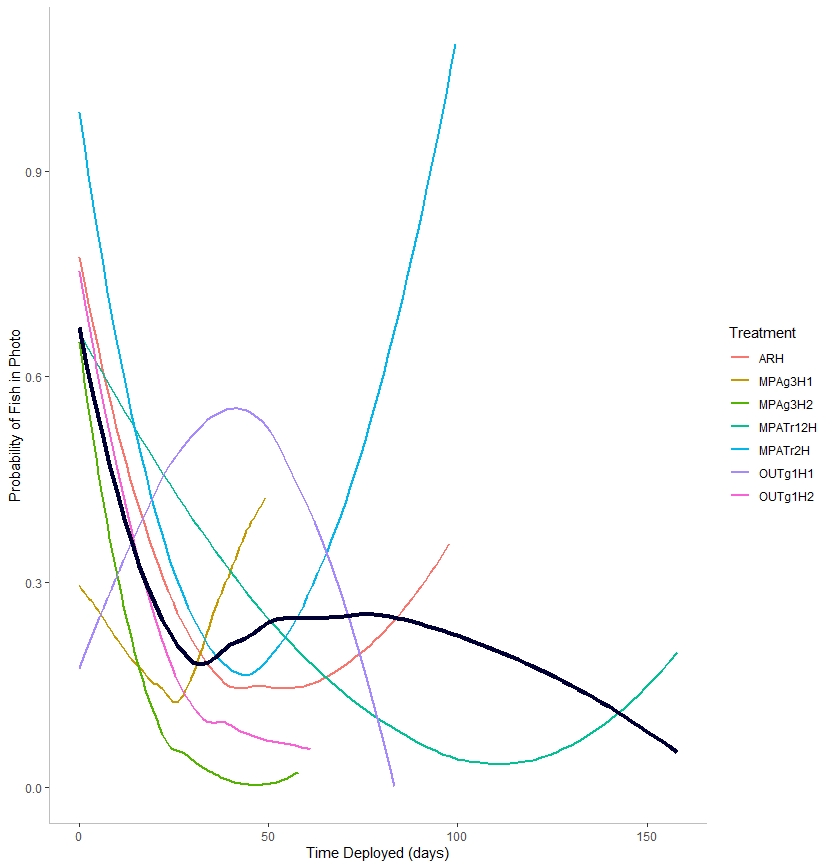

Supplement: S3 Fig — Lines were fitted with the “loess” local polynomial regression fitting function with span = 1 from R package ggplot2 (Wickham 2009). The dark line represents the smoothed results of all sites shown on this graph. Not only do fish detections decline consistently at most sites due to biofouling of the camera lens over the first month, model accuracy is clearly impaired past this point at certain sites, leading to a false increase in fish detection probability for some cameras (e.g., note the erroneously high probabilities around 80–100 days for MPATr12H, shown in blue). (JPEG) [file pone.0263377.s003.jpeg]

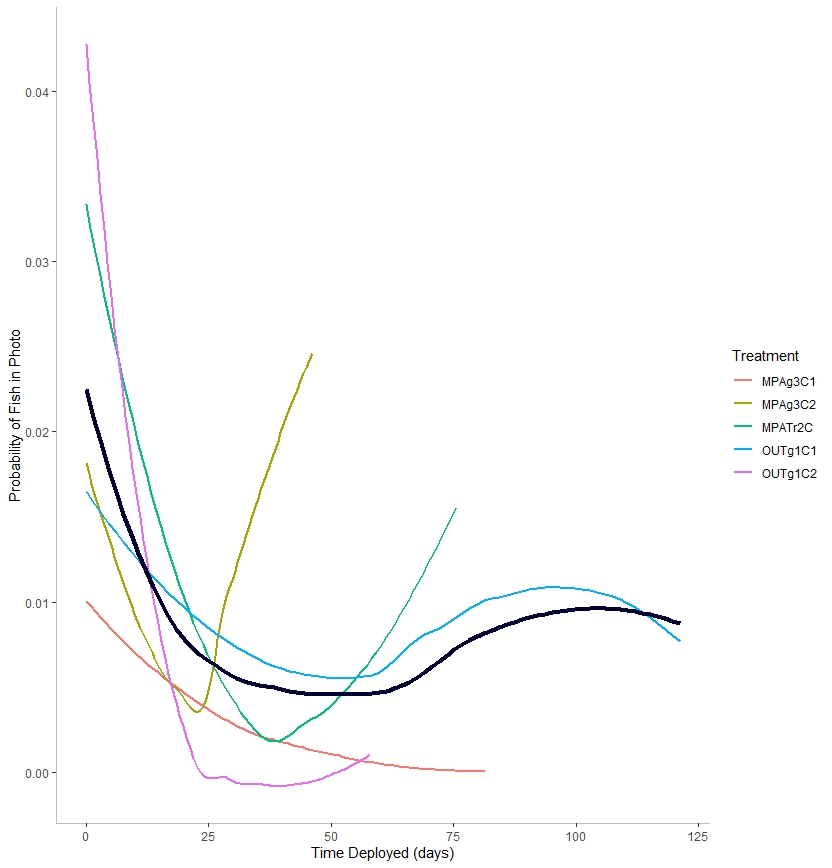

Supplement: S4 Fig — Lines were fitted with the “loess” local polynomial regression fitting function with span = 1 from R package ggplot2 (Wickham 2009). The dark line represents the smoothed results of all sites shown on this graph. Note that fish detection probabilities are lower at grass sites, relative to reef/halo sites, and the y-axis is scaled appropriately. After the first month, fish detection probabilities erroneously rise at a couple sites, likely reflecting reduced accuracy in the ResNet-50 model’s image classifications as a result of biofouling. (JPEG) [file pone.0263377.s004.jpeg]

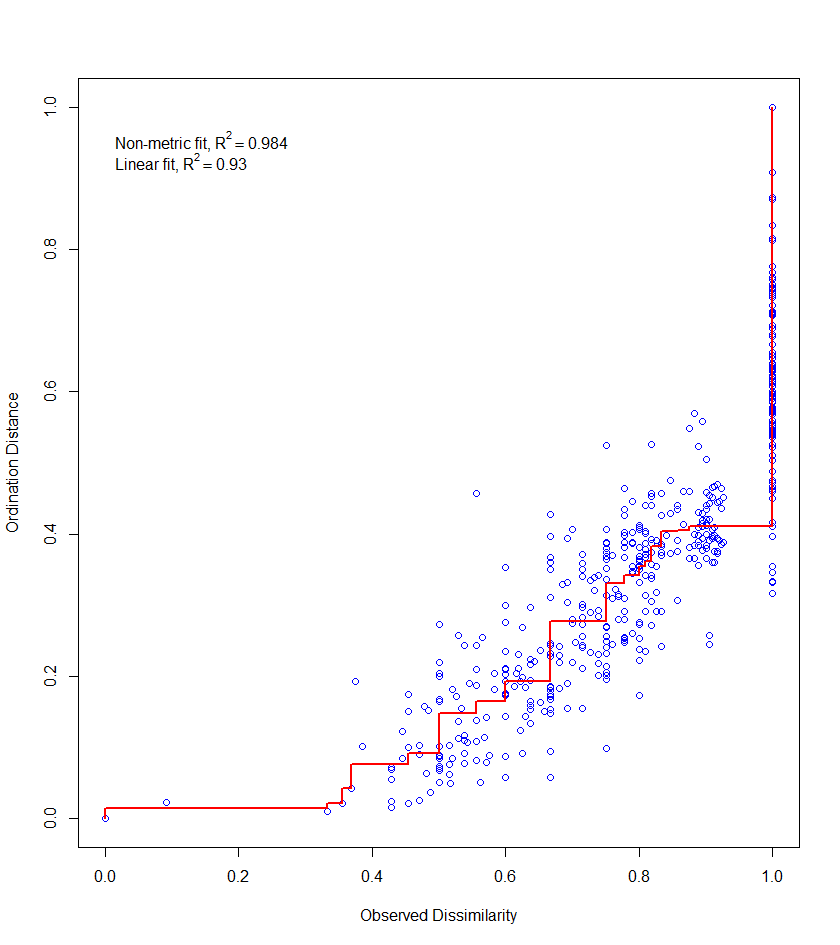

Supplement: S5 Fig — Created using the stressplot() function in R package vegan (Oksanen et al,. 2018) [42]. (PNG) [file pone.0263377.s005.png]
